# Oxidation of Sulphur pollutants in model and real fuels using hydrodynamic cavitation

**DOI:** 10.1016/j.ultsonch.2023.106405

**Published:** 2023-04-14

**Authors:** Peter Delaney, Varaha P. Sarvothaman, Sanjay Nagarajan, David Rooney, Peter K.J. Robertson, Vivek V. Ranade

**Affiliations:** a School of Chemistry and Chemical Engineering, Queen’s University Belfast, Belfast BT9 5AG, UK; b Clean Combustion Research Center, King Abdullah University of Science and Technology, Thuwal 23955-6900, Saudi Arabia; c Sustainable Environment Research Centre, University of South Wales, Pontypridd CF37 1DL, UK; d Bernal Institute, University of Limerick, Limerick, Ireland

**Keywords:** Oxidative desulphurization, Hydrodynamic cavitation, Fuels, Catalysts, Oxidants

## Abstract

Hydrodynamic Cavitation (HC) offers an attractive platform for intensifying oxidative desulphurization of fuels. In the first part of this work, we present new results on oxidising single ring thiophene in a model fuel over the extended range of volume fraction of organic phase from 2.5 to 80 v/v %. We also present influence of type and scale of HC device on performance of oxidative desulphurization. Further experiments revealed that oxidising radicals generated in-situ by HC alone were not able to oxidise dual ring thiophenes. External catalyst (formic acid) and oxidising agents (hydrogen peroxide, H_2_O_2_) were therefore used with HC. Based on our prior work with acoustic cavitation (AC), the volumetric ratios for H_2_O_2_ and formic acid were identified as 0.95 v/v % and 6.25 v/v % respectively. The data of oxidation of dual ring thiophenes with n-dodecane and n-hexane as model fuels and typical transport fuels (diesel, kerosene, and petrol) using these oxidant and catalyst is presented. The observed performance with HC was compared with results obtained from a stirred tank and AC set-up. The presented data indicates that HC is able to intensify oxidation of sulphur species. The presented results provide a sound basis for further developments on HC based oxidative desulphurization processes.

## Highlights

• Thiophene removal using HC was effective up to fuel volume fraction of 80%
• Presented influence of type and scale of HC devices on desulphurization performance.
• External oxidant-catalyst is a necessary for dual-ring thiophene desulphurization.
• Quantified oxidation performance of HC with stirring and AC based processing.

## Nomenclature

ΔP: Pressure drop (kPa)
add: Additives (-)
aq: Aqueous phase (-)
B.pt: Boiling point (°C)
C_0_: Initial concentration (ppm)
d_t_: Throat diameter of vortex diode (mm)
Dev 1: Vortex-diode device with d_t_ = 6-mm
Dev 2: Orifice device with d_t_ = 5-mm
Dev 3: Vortex-diode device with d_t_ = 12-mm
En: Energy Expense (J)
n: Number of passes through cavitation device (-)
org: Organic phase (-)
PI: Pressure indicator (-)
T: Temperature (°C)
v: Volume (mL)
v/v: volume/volume (mL/mL)
V: Valve (-)
V_f_: Volume fraction (-)
w_,DBT, rem_: mass of DBT removed (mg)
X: Conversion (%)
y: Extent of difference in reactivity of intermediates (-)
Y: Sulphur removal per energy expense (mg/J)
ΔE/R: Activation energy for pollutant (K)

**Acronyms**
AC: Acoustic Cavitation
ACN: Acetonitrile
ADS: Adsorptive Desulphurization
BDS: Biodesulphurization
CH_3_COOH: Acetic Acid
DBT: Dibenzothiophene
EDS: Extractive Desulphurization
FLD: Fluorescence detection
H_2_O: Water
H_2_O_2_: Hydrogen Peroxide
HC: Hydrodynamic Cavitation
HCOOH: Formic Acid
HDS: Hydrodesulphurization
MeOH: Methanol
OH•: Hydroxyl radical
SO_x_: Sulphur oxides
SRR: Sulphur removal rate
UAOD: Ultrasound Assisted Oxidative Desulphurization
UV: Ultraviolet

**Greek Symbols**
β: Ratio of flow rate through cavitation device and holding tank volume (s^-1^)
*γ*: Parameter indicating positive or negative deviation for changing per-pass constant (-)
*η*: Conversion per unit additive volume (%/mL)
θ: Organic fraction (%)
θ_add_: Volumetric ratio of additives (-)
∅: Per-pass removal constant (-)
$\emptyset_{0}$: Per-pass removal coefficient (-)
μ: Viscosity (mPa.s)
$w_{s0}$: Weight of sulphur in reaction mixture (mg)

## 1 Introduction

Reduction of the environmental impact of Sulphur emissions (SO_x_) from combustion of transport fuels is a major focus for governing bodies aiming to ensure a high standard of air and water quality. Recently the international maritime organisation (IMO) restricted the Sulphur content of shipping vessel fuels to 0.5 wt% globally and 0.1 wt% in restricted areas (known as IMO 2020 Global Sulphur Cap) [1]. This is a significant drop in an industry which utilises small capacity docking refineries where bringing down Sulphur content using hydro desulphurization (HDS) becomes expensive. In fact, the economic impact of these regulations has brought great concern for refineries and independent trading ship owners with less complex equipment [2]. To address these concerns, alternative technologies to HDS are highly desirable to minimise capital and operating expenditure needed for implementing desulphurization [3].

The four most researched alternative desulphurization technologies suitable for smaller capacities are adsorptive desulphurization (ADS), bio desulphurization (BDS), extractive desulphurization (EDS) and oxidative desulphurization (ODS). BDS is restricted for utilisation for treatment of transport fuels due to its long residence time [4], whilst ADS and EDS are often combined with ODS to overcome the individual limitations of each process. In the ODS process, the sulphur containing species are oxidised to corresponding sulphone molecules, which are then extracted by a suitable extractant such as methanol/acetonitrile. Oxidation of aromatic refractory Sulphur species alters the compounds’ polarity allowing for easier separation by improving selectivity towards adsorbents and polar solvents [5]. Unlike the HDS process, the ODS process suffers from a disadvantage that it is accompanied by some loss of carbon which goes to extractant phase along with sulphones. However, ODS exhibits favourable chemistry compared to HDS as the Sulphur compounds, which are resistant to hydrotreatment are more susceptible to oxidation. ODS process is also amenable to decentralised implementation unlike HDS and thus offers the potential for smaller scale applications such as desulphurisation of biofuels.

The ODS typically involves two immiscible phases: an organic fuel with Sulphur containing species and an aqueous polar phase containing oxidant and/or catalyst [6]. The performance of such multiphase ODS systems is often limited by effectiveness of contact between two phases containing Sulphur species and oxidising species. Hydrodynamic cavitation which generates strongly oxidising species like hydroxyl radicals in-situ and intense shear which enhances interphase mass transfer and mixing offers an attractive technology for implementing ODS [7], [8]. Cavitation is the phenomenon of formation, growth, and collapse of cavities (microbubbles). Collapsing cavities generate intense shear and highly localized temperature and pressure, which, in turn, generate strongly oxidative species. Cavitation can be realized either ultrasonically or hydrodynamically [9], [10]. Acoustic cavitation (AC) is energy-intensive and difficult to scale up. Hydrodynamic cavitation (HC), however, can be scaled up relatively easily and has significantly better energy efficiency compared to AC [11].

HC based ODS has started to receive increasing attention. Recent works have focused on utilising water-fuel based systems for treating various thiophene containing model fuels, diesel [7], [8] and pyrolysis oil [12]. These studies, however, are limited to organic volume fractions ≤10% and single ring aromatic thiophenes found in lighter fuel fractions such as gasoline. In the first part of this work, we conducted a parametric study for thiophene oxidation in a water-fuel HC set-up, assessing the influence of initial concentration, temperature, pressure drop across the device and volumetric fraction of the organic phase. To our knowledge, performance of ODS with a continuous organic phase has not been investigated for water-fuel cavitation systems. Extending the volumetric volume fraction up to 80% allows for comparison with fuel volumes utilised in conventional additive based ODS based processes. Possible use of aeration to enhance oxidation was also evaluated. Additionally, systematic comparison of type and scale of cavitation device has also been completed for the first time for ODS. This work compares linear and swirling flow devices (orifice versus vortex diode) and varying scales of geometrically similar vortex diode. As shown earlier it was not possible to oxidise dual ring thiophene species such as dibenzothiophene (DBT) with water alone. The second part of this work therefore investigated additive based HC, for which only a small number of publications have reported so far [13], [14], [15].

The additive-based study was conducted using optimised volumetric fractions of hydrogen peroxide oxidant and carboxylic acid catalyst, identified as 0.95 v/v % H_2_O_2_ and 6.25 v/v % HCOOH using our previous AC investigation [16]. HC experiments were completed with model fuels (acetonitrile, dodecane and hexane) to consolidate the design of experimental configurations at various process capacities and scales of vortex diode as cavitating device. These conditions were then applied to actual fuels. HC studies including actual fuels and additives are very limited and we have discussed key considerations for designing HC set-ups with continuous organic media. The ODS performance at three scales of vortex-based cavitation device (vortex diode) and different methods of additive loading (initial loading vs stepwise addition) was investigated. Experiments with actual fuels were then completed and compared using AC and stirred set-ups.

We have compared HC device type using per pass methodology developed in our previous work [17] which was found to be useful for characterisation of HC ODS set-ups of different capacities and scale of cavitation device by allowing for accurate comparison of degradation and/or oxidation profiles. We have focused on not utilising additional treatment steps such as extraction or adsorption. This allows us to accurately assess the impact of cavitation on oxidation. The results presented here will be useful for harnessing hydrodynamic cavitation for intensifying ODS.

## 2 Materials and methodology

### 2.1 Materials

Acetonitrile (ACN) (≥99.9%, Sigma Aldrich), n-dodecane (99%, Thermo Scientific) and n-hexane (≥99%, Sigma Aldrich) were used as model fuels. Actual fuels used in this study: diesel, kerosene, and petrol were purchased from a local fuel station. Model Sulphur (S) pollutants utilised are Dibenzothiophene (DBT) (98%, Sigma-Aldrich) and Thiophene (>98%, Sigma Aldrich). Appropriate quantities of pollutants were added to the organic phase for the initial concentrations investigated throughout the study. Additives included were 30 v/v % Hydrogen Peroxide (H_2_O_2_) (Scientific Lab Supplies), Acetic acid (CH_3_COOH (99%, Sigma Aldrich) and Formic Acid (HCOOH) (99%, Sigma Aldrich) which are used as oxidant and acid catalysts respectively. ACN was also used as the mobile phase for HPLC-UV-FLD analysis [18].

### 2.2 Experimental set-ups

HC experiments were conducted in 3 different configurations (Table 1) to investigate evaluation of influence of type and scale (or operating capacity) of cavitation device. Experiments were carried out using linear (orifice device) and swirling flow (vortex diodes) HC devices. Configurations 1 – 3, which were used for HC studies with additives all featured a vortex diode as a cavitation device. The scale of device was indicated by their throat diameter (d_t_), being: d_t_ = 6 mm (Dev 1, vortex diode, stainless steel), d_t_ = 5 mm (Dev 2, orifice, stainless steel) and d_t_ = 12 mm (Dev 3, vortex diode, Perspex). Additional details on the geometry and flow characteristics of these cavitation devices can be found in Simpson and Ranade [19]. Details of experimental set-up and performance of HC in terms of degradation of organic pollutants may be found in earlier works of Ranade and co-workers [9], [10].

**Table 1:** Different experimental configurations used in this work.

Configuration	Holding Tank	Cooling Method	Cavitation Device	Flow rate @ ΔP = 200 kPa (LPM)	Fuel	Fuel Volume (mL)	Sulphur Pollutant	H_2_O (v/v %)	H_2_O_2_ (v/v %)	HCOOH (v/v %)
1	10 L Stainless steel tank	Stainless steel coil	6 mm vortex diode (Dev 1)	4.5	Dodecane	5500	Thiophene	10–90	–	–
1	10 L Stainless steel tank	Stainless steel coil	5 mm orifice (Dev 2)	4.5	Dodecane	5500	Thiophene	10–90	–	–
2a	2.5 L Baffled beaker	Stainless steel coil	6 mm vortex diode (Dev 1)	4.5	Dodecane, Hexane	2650	DBT	–	0.95	6.25
2b	2.5 L Baffled beaker	Glass cooling coil	6 mm vortex diode (Dev 1)	4.5	Dodecane, Hexane	2650	DBT	–	0.95	6.25
2b	2.5 L Baffled beaker	Glass cooling coil	12 mm vortex diode (Dev 3)	18	Diesel, Dodecane	3500	DBT	–	0.95	6.25
3	10 L Stainless steel tank	Stainless steel coil	12 mm vortex diode (Dev 3)	18	Diesel, Kerosene, Petrol	8352.9	DBT	–	0.95	6.25

All experiments with Thiophene as model pollutant were completed with Configuration 1. This set-up utilised a Grundfos CM 1 – 5 self – priming pump (2900 rpm, 50 Hz, single phase), and the piping material comprised stainless steel and plastic pipes (after checking compatibility with dodecane). Experiments to quantify the effect of pressure drop, volume fraction and initial concentration were conducted with temperature maintained at T = 20 ± 2 °C, using cooling water circulated through a stainless-steel cooling coil connected to mains water and placed in the holding tank. To evaluate the effect of temperature, an experiment was conducted in a non-isothermal mode. The highest temperature observed was 45 °C and observations of temperature were made during each sample collection. As for the effect of organic fraction, the measured quantities of organic and aqueous phases were mixed in a desired ratio and circulated through the cavitation device. Pressure drop (ΔP) was adjusted by controlling flow through the bypass and cavitation loops using the installed valves.

Experiments for the device scale – up involved switching device Dev 1 with Dev 3 on the experimental setup. An orifice device (Dev 2) with a similar flow curve as that of device Dev 3 was employed in the study. Ensuring similar flow curve between the vortex diode and the orifice allows for an objective comparison of device types as they exhibit similar power consumption. This was validated in a study by Sarvothaman *et al.* comparing five different device types [10]. For the effect of aeration, Dev 2 was selected as it possessed the largest scope for improvement. An aerator with maximum capacity of 35 LPM was installed with a single tube sparger to evenly disperse the bubbles in the tank.

Experiments with additives were conducted with Configurations 2 and 3. The holding tank was a glass beaker placed in an ice bath for temperature control. Configurations 2a and 2b involved swapping the holding tank from a 10*L* stainless-steel open tank to 2.5L baffled glass beaker to minimise evaporation of fuels. Configuration 2a included a stainless-steel cooling coil with overhead mixer and careful placement of the pump inlet/outlet in attempt to maximise interfacial mixing and circulation. Configuration 2b utilised a glass cooling coil with the pump inlet placed inside the coil to maximise contact between the medium and the coil. The beaker was placed on a magnetic stirring plate and the inlet was given sufficient clearance to the bottom of the vessel to ensure the solution could be stirred properly. The pump outlet was placed further up the holding tank to maximise mixing. Both configurations 2a and 2b utilised a centrifugal pump (Pedrollo 4CR 80-n) with variable frequency drive (VFD) to restrict deviation of the flow rate through the cavitation device. Configuration 2a and 2b did not include a bypass line and ΔP was set by the internal controller. A valve was also installed for manual control of the flow rate through the device. Initially Dev 2 was installed on both 2a and 2b, after which Dev 3 was installed on configuration 2b to improve interfacial mixing. The nominal flow rate of Dev 3 was higher than that of Dev 1 (18 LPM, as against 4.5 LPM) and therefore the stirring plate was no longer required when Dev 3 was installed. It was obvious from visual inspection that a well-mixed emulsion had formed. Stainless steel piping was used. Configuration 3 utilises a 10 L stainless-steel tank with stainless steel cooling coil and overhead mixing. A centrifugal pump (Model 3C R80, Pedrollo) was installed on configuration 3, with a gravity fed inlet for bottom suction from the holding tank ensuring both polar and organic phases are circulated through the cavitating line. This set-up included a bypass line to control the pressure drop across the vortex diode and could only be operated with Dev 3 because of the higher flow rates. The data presented for concentration profiles is presented with respect to number of passes through the cavitation devices. One pass corresponds to residence time in the closed loop (ratio of volume and flow through cavitation device). Total number of passes, n can be related to elapsed time, t as: n = t/τ where τ is a residence time.

**Fig. 1:**
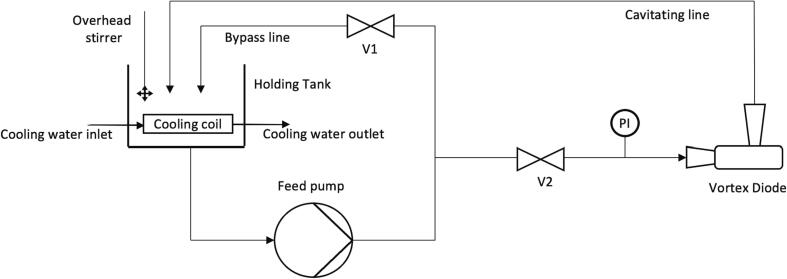
Schematic of HC set-ups.

All set-ups utilised pre-calibrated pressure gauges (Thermosense direct) installed before the cavitation device to monitor pressure (also installed on bypass line in configuration 3). The temperature was maintained at T = 20 ± 2 °C in all set-ups, except for experiments investigating the impact of non-isothermal operation, where the temperature was varied between 20 and 45 °C. Pressure drop was maintained at ΔP = 200 kPa based on the results of our previous study [16]. This ensured that there was not excessive turbulence in the holding tank and the majority of flow passed through the cavitating line rather than the bypass line (for Configurations 1 and 3). A general schematic is presented in Fig. 1.

Draining and venting of the HC rigs was important to ensure that quantities of additives remained constant and no residuals remaining in the rig. In all circumstances the rig was drained, and compressed air was circulated throughout the system, this ensured that any remaining fluid was expelled. In HC experiments with additives, the system was flushed with n-hexane to reduce any residual water remaining in the set-up and the drainage/venting procedure was repeated. The pump was then primed with the required medium and circulated for an appropriate amount of time, an initial sample (t = 0 min) was then collected, and subsequently oxidant/catalysts were added into the tank near the inlet suction. For additive based experiments the volumetric ratio of H_2_O_2_ and HCOOH were determined based on a parametric study from our previous work using AC [16]. These were set at 0.95 v/v % H_2_O_2_ and 6.25 v/v % HCOOH, and were added in HC experiments by various dosing techniques (initial dosing versus staggered dosing). These techniques of additive loading are discussed in the appropriate sections below, where initial dosing/loading refers to all additives being loading at t = 0 min. Stepwise addition indicated that the addition of the same quantity of additives was carried out at dedicated intervals (i.e.*,* the total amount of additives/number of intervals and subsequently added at the pre-determined intervals).

In addition to HC experiments, to compare with previous studies, AC experiments with diesel, kerosene, and petrol were completed using a Sonics Vibra-Cell 500 W VCX 500 with horn tip (diameter = 13 mm) submerged 1 cm into the medium. Ultrasonic parameters were determined within our previous work and set at: frequency = 20 kHz, duty cycle = 50% (3 s ON / 3 s OFF) and acoustic amplitude = 80% [16]. The reactor vessel was a 50 mL jacketed beaker connected to mains water which circulated through the beaker jacket and maintained temperature at T = 20 ± 2 °C. All AC experiments had a total volume (v_total_) = 40 mL. Stirred experiments were carried out with a MS-H-Pro + stirring plate with suitably sized stirring bar for two volumes – 40 mL, and 1000 mL (agitation speed set at 350 RPM and 1000 RPM, which ensured no splashing of fuels). No additional cooling was required for stirred set-ups and temperature also remained at 20 ± 2 °C. Temperature was monitored during sampling for all experiments. Both the stirred and AC experiments were carried out for a total time of 30 min.

### 2.3 Analytical techniques

For HC based experiments with water, a sample of the emulsion was collected at regular time intervals. After approximately 10 min, there was a distinct separation of the aqueous and organic phases. The organic layer was collected and analysed using gas chromatography (GC) equipped with a FID detector. The method conditions were set as: initial temperature = 60 °C, ramp rate = 20 °C/min and final temperature = 250 °C. The organic fractions ranged from 2.5 to 80 v/v % in the experiments. In the case of the lean organic fraction (2.5%), the sample volume collected was 250 mL into a standard flask, the ‘organic’ layer was centrifuged at 15,000 rpm for 15 min. For experiments operated with higher organic volume fractions, this procedure was not required and 5–10 mL was collected and centrifuged in the same manner. Following centrifugation, 1 mL of sample was analysed.

For experiments with additives, samples were collected at regular intervals with 2 mL of fuel withdrawn in a 2 mL Eppendorf tube and centrifuged for 7 min at 15,000 rpm. In HC experiments, the organic phase without additives was added to and circulated in the set-up for 10 min, to achieve complete mixing, after which a sample was collected and labelled as t = 0 min. Initial samples for AC and stirred experiments were taken from the prepared stock solutions, whereas 2 mL was collected at regular intervals (at t = 10, 20 and 30 min). After centrifugation, 1 mL of the organic phase was transferred to a sample vial and analysed using High performance liquid chromatography – UV – fluorescence detection (HPLC-UV-FLD). Complete phase separation was confirmed by visual confirmation and careful pipetting. A Kinetex 5 µm C18 column was used for this purpose. Mobile phases of solvent A – pure acetonitrile and solvent B – deionised water was prepared to mobilise the samples through the stationary phase. A linear flow gradient from 100% A, 0% B (run-time = 0 min) to 90% A, 10% B (run-time = 8 min) gave the most effective separation between components. No additional run-time was provided. A sample volume of 5 μL was injected into the column using an autosampler. The samples were analysed using a PDA-100 Photodiode Array Detector. The UV detector was set at a wavelength of 287 nm and the mobile phase flow rate was 0.5 mL/min.

### 2.4 Evaluation of per pass removal factor

For comparing performance of different devices and scales, we used the per-pass model for interpreting experimental results obtained with devices: Dev 1 and Dev 3 (swirling flow) and Dev 2 (linear flow) [11]. The per-pass approach allows systematic comparison over different flow rates, scales and volume of holding tanks. Following our previous work for estimating the degradation performance of organic pollutants in water [17], the overall behaviour of such a batch experimental set-up can be modelled as:

(1) $$V\frac{\mathrm{dC}}{\mathrm{dt}}=-Q\emptyset C$$

Here C is the concentration of sulphur species in the organic phase (and emulsion), V is the volume of entire holding tank (aqueous and organic phase), Q is a flow rate through cavitation device and ∅ is a per – pass removal factor. If the value of ∅ is assumed to be constant over a range of concentration and time, this value can be estimated using experimental measurement of concentration of pollutant as a function of time and Eq. (1) as:

(2) $$C=C_{\mathrm{in}}e^{-\beta \emptyset t}$$

where β is a ratio of flow rate through cavitation device and holding tank volume (Q/V, s^−1^). The product of βand time indicates number of passes, n, through the cavitation device for a batch system:

(3) n=βt

The model presented so far describes the process under isothermal conditions. For non-isothermal operation, the model can be readily extended by relating per pass degradation factor to activation energy as [17]:

(4) $$\emptyset =\emptyset_{0}e^{-\Delta E/RT}$$

Eq. (2) is then re-written as:

(5) $$C=C_{\mathrm{in}}e^{-\beta \emptyset_{0}\int e^{-\Delta E/RT}dt}=C_{\mathrm{in}}e^{-\emptyset_{0}\int e^{-\Delta E/RT}dn}$$

It was observed that HC experiments with additives were not described adequately well using the constant per-pass factor model. The extended per-pass factor with varying per-pass removal factor [9] was therefore used to interpret experiments of HC with additives as:

(6) $$ln\left(\frac{C}{C_{0}}\right)=-\emptyset_{0}\left[\frac{{\left(1+\gamma \emptyset_{0}n\right)}^{y-1}-1}{\emptyset_{0}\left(y-1\right){\left(1+\gamma \emptyset_{0}n\right)}^{y-1}}\right]$$

The equation contains three parameters: initial per-pass degradation factor, ∅_0_; γ which indicates the sign of change of per pass degradation factor with number of passes and parameter y which indicates the severity of the change. When the value of parameter, y is zero, the model will reduce to the simple per-pass removal factor as for the system with no additives (water only).

## 3 Results and discussion

The first section investigates thiophene removal from dodecane using HC subjecting a fuel-water mixture to hydrodynamic cavitational treatment. Systematic experiments were performed to quantify the effect of various operating conditions (initial concentration, temperature, organic fraction) and identify the optimal device parameters (pressure drop, scale, type). Experiments were initially conducted with single ring thiophene and then with dual ring aromatic species without using any external oxidant or catalyst.

### 3.1 Removal of single ring thiophene

The studies reporting the use of HC ODS, by the approach of mixing fuel and water have considered either more than 10,000 ppm [12] or low initial concentrations up to 300 ppm [7], [8]. Initially we carried out experiments with an intermediate range of initial concentrations - from 1000 to 7500 ppm of thiophene (380 to 2850 ppm S). Experiments were typically conducted for 50 or 100 passes and samples were collected multiple times. Within the studied range of initial concentrations, the per – pass removal factor, ∅ was found to be independent of initial concentration. The value of ∅ was obtained as 25.7 × 10^−3^. The subsequent experiments were performed with an initial concentration of 2500 ppm thiophene.

**Fig. 2:**
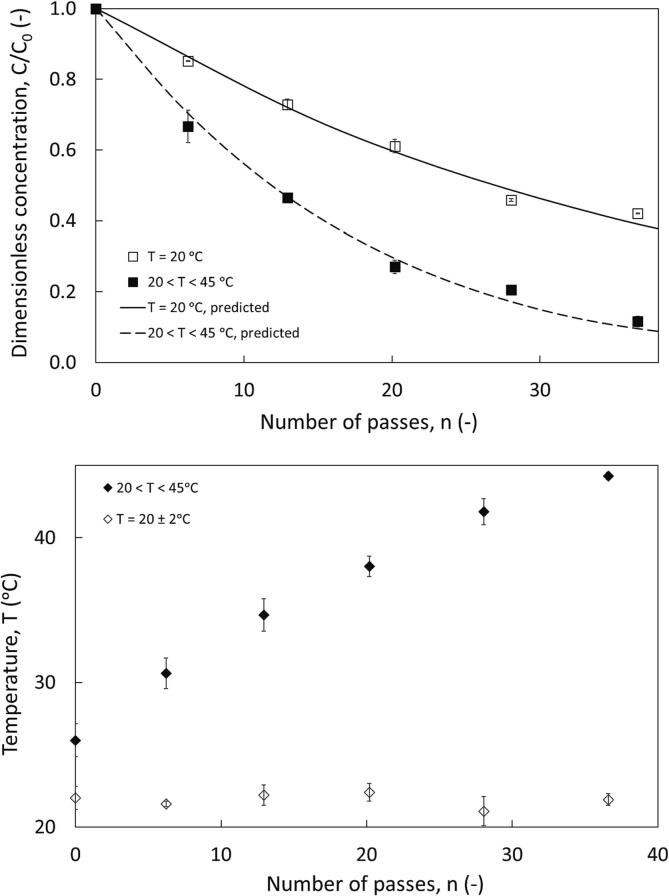
a) Influence of temperature on thiophene removal from dodecane, configuration 1, Dev 1, v_org_ = 2.5%, C_0, thiophene_ = 2500 ppm, ΔP = 200 kPa, ∅ × 10^3^ for predicted curves = 32.2 (isothermal) and 44.7 (non-isothermal) and b) measured temperature profile.

Influence of operating temperature on observed removal performance was then investigated. In the case of isothermal experiments, the temperature was maintained at T = 20 ± 2 °C. For non-isothermal experiments, the initial temperature was recorded as T = 20 ± 2 °C also. Circulation through HC device leads to increase in temperature with increase in number of passes which eventually plateaus. The temperature was monitored as a function of number of passes. The sulphur removal was improved for the non-isothermal case (Fig. 2).

The net removal was increased by 1.5 times when a non-isothermal operation was performed (89% and 58% in 36 passes with non-isothermal and isothermal operation respectively). The final S concentration are 110.0 and 399.3 mg/kg with non-isothermal and isothermal operation respectively. Pressure drop is one of the primary device parameters to be investigated for a cavitation-based application. In a cavitation device, the inception of cavitation depends on the physicochemical characteristics of the feed (in this case emulsion of dodecane – water), operating temperature and dissolved gases. Suryawanshi et al. reported inception occurs < 100 kPa in water-fuel mixtures and therefore experiments were carried out at 100, 200 and 300 kPa [7], [8]. The 200 kPa condition was chosen as the pressure drop for further investigation based on estimated per-pass removal, and this served as a condition for comparison with devices Dev 2 and Dev 3.

#### 3.1.1 Influence of scale and type of devices

**Fig. 3:**
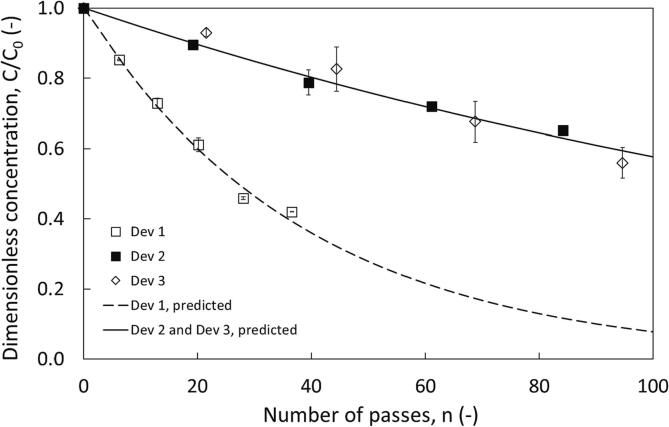
Influence of device type and scale on thiophene removal from dodecane, configuration 1 v_org_ = 2.5%, T = 20 ± 2 °C, C_0, thiophene_ = 2500 ppm, ΔP = 200 kPa, ∅ × 10^3^ for predicted curves = 32.2 and 6.9 for Dev 1 and (Dev 2 and Dev 3) respectively; predicted using Eq. (2).

Vortex based cavitation devices have been utilised for HC-ODS based studies with water. Geometrically similar devices were considered by Uebe et al. [12] (similar to Dev 3) while Suryawanshi et al. [7], [8] compared the vortex diode with an orifice. However, systematic comparison of device scale and device type have not been reported yet for ODS. Systematic comparison of hydrodynamic cavitation devices has been completed for continuous wastewater treatment. However such data is not currently available for multiphase process like ODS. Comparison of linear and vortex based devices allows for comparison with other publications. Sarvothaman et al. demonstrated increasing device scale (and associated flow rate) reduced the per pass removal factor. However this enhanced flow rate may assist with mixing the aqueous and organic phases in ODS. The per pass factor model described herein allows for comparison of cavitation devices operating at different flow rates. In order to achieve this, comparison of Dev 1 and Dev 3 (geometrically similar devices of the vortex – diode type) was employed. The ratio of characteristic dimension/throat of device varied by a factor of two. Thus, the nominal capacity varied by a factor of four. Comparison of the scale of swirling flow devices (Dev 1 and Dev 3) and swirling versus linear flow, where an orifice (Dev 2), which operated with a similar flow curve as Dev 3, (ensuring a similar power consumption when operated at identical pressure drop conditions) are compared in Fig. 3.

The sulphur removal varied considerably for the devices Dev 1 and Dev 3, which was not surprising. The per-pass removal coefficient was 4.7 times superior for device Dev 1 when compared to that of Dev 3 (with four times more flow rate than Dev 1). It was observed that the sulphur removal was similar and could be characterised by the same per-pass removal coefficient’s value. Similar behaviour with increasing scale of device has been reported for the application of these devices for water treatment [9], [10], [17]. Appropriate strategies like numbering-up may therefore have to be employed for scaling up HC based ODS with maintaining same per-pass removal factor.

#### 3.1.2 Influence of aeration

Aeration is a chemical free means to improve performance of cavitation-based processes. For cavitation based wastewater treatment processes, studies have reported the enhanced removal of organic pollutants with aeration [17], [20]. Aeration can enhance degradation in cavitation-based processes by providing additional nuclei for cavity formation, enhancing the extent of cavitation. Sarvothaman et al. reported a nearly 2.5 time increase in the degradation of acetone in water with aeration of 1 vvm [17]. Aeration in cavitation based ODS systems is a relatively unstudied enhancement technique. Cako et al. report that combining aeration with acoustic cavitation could enhance removal of thiophenic species in naptha from 6.0%–42.4% at an air flow rate of 0.5 dm^3^/min, suggesting utilisation of aeration as a technique for increasing cavity nucleation is viable for ODS [21]. In the current study, aeration of 1.9 vvm was used with device Dev 2 (orifice based device) and the per-pass removal was found to be enhanced by 3.6 times. A range of flow rates could not be tested due to limitations of the equipment. Aeration is a viable means for improving HC ODS. A summary of the obtained per-pass removal coefficients obtained at different operating conditions is provided in Table 2.

**Table 2:** Comparison of estimated per-pass removal factors for various investigations in HC study.

Device	v_org_ (%)	T (^o^C)	ΔP (kPa)	Thiophene – C_0_ (ppm)	Aeration (vvm)	φ × 10^3^ at 300 K (–)
Dev 1	2.5	20	200	1000 < C_0_ < 7500	0	32.2
Dev 1	2.5	20	100	2500	0	61.4
Dev 1	2.5	20	300	2500	0	11.2
Dev 1	10	20	200	2500	0	32.0
Dev 3	2.5	20	200	2500	0	6.9
Dev 2	2.5	20	200	2500	0	6.9
Dev 2	2.5	20	200	2500	1.9	24.6

#### 3.1.3 Influence of volume fraction of organic phase

The operating conditions investigated so far have utilised similar volume fractions reported elsewhere. Suryawanshi et al., reported that increasing the organic volume fraction from 2.5 to 10 v/v % had a negative impact on the extent of thiophene removal for all tested model fuels, presenting a reduction of thiophene removal in diesel from 74 % to 45 %, with similar trends shown for n-octane, n-octanol, and toluene. However, the net removal as a function of volume fraction indicates a larger mass of Sulphur is removed at the higher volume fraction for diesel (54 mg removed at 10% v/v vs 22.2 mg at 2.5% v/v [assuming a volume of 12L]), and octanol (72 mg removed at 10% v/v vs 27.6 mg at 2.5% v/v). Whereas net removal for n-octane remains approximately equal (22.8 mg removed at 10% v/v vs 23.1 mg at 2.5% v/v). Uebe et al. 2021 showed that increasing the oil fraction from 2.5 to 7 v/v % had a positive impact of removal but concluded that HC only positively impacted the process from 5.5 to 6 v/v % [12]. Our results, however, show that increasing the volume fraction from 2.5 to 10 v/v % did not significantly impact S removal or the per pass factor. This potentially could be caused by differences, in volume, set-up or the organic phase properties of the set-ups and solvents utilised in this study. However, the higher removal is beneficial as more pollutant is being removed from the fuel at higher organic volume fractions. We investigated increased volume fractions (>10 % volume fraction of organic) here. The organic phase volume was increased up to 80 v/v %. These experiments with continuous organic phase were also a better representation of additive containing ODS systems, which typically had organic volume fractions exceeding 90 v/v % [16]. Organic solvents are less ideal than water for cavitation-based processes, typically exhibiting higher vapour pressures and lower surface tension than water, leading to lower collapse temperatures and forming less radicals. Additionally the bubble composition in the organic-aqueous mixture may contain organic compounds which may dissociate to form scavenging radicals [22]. Investigations of a dispersed aqueous phase in oil–water cavitation systems focus on emulsification [23], whereas dispersed water can be utilised in emulsion fuels to improve combustion efficiency and reduce combustion peak temperature reducing GHG emissions [24], which can be realised using HC [25].

**Fig. 4:**
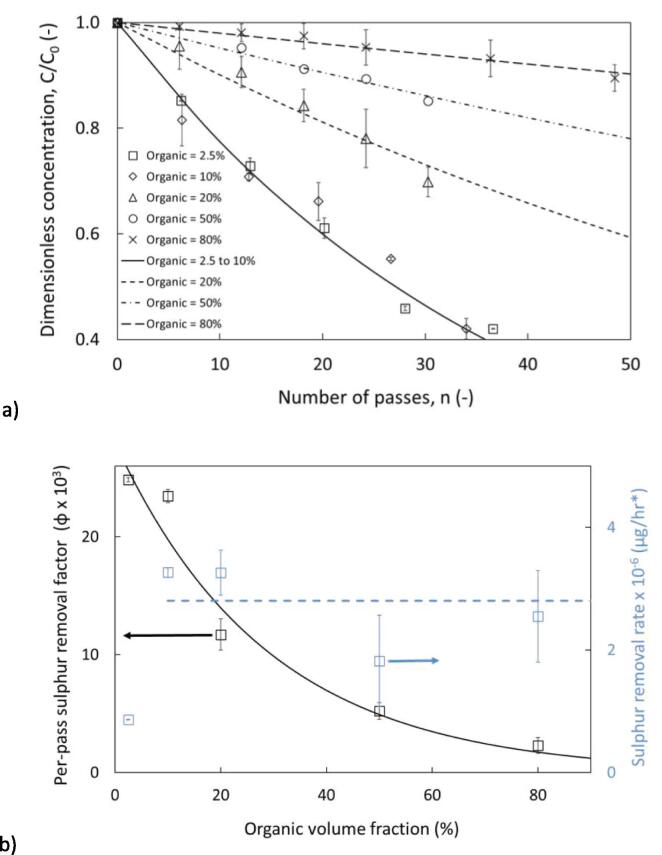
Influence of organic volume fraction on a) dimensionless concentration removal profile of thiophene, b) per-pass removal and sulphur removal rate. Experiments performed on configuration 1, Dev 1, C_0, thiophene_ = 2500-ppm, T = 20 °C @ ΔP = 200 kPa, V_tot_ = 5.5 L [8].

The results showed that increasing the organic volume fraction had a detrimental impact on the concentration profiles for volume fraction > 10% (Fig. 4a). Reducing the volume of water may reduce the amount of Sulphur that can be extracted by transfer to the aqueous phase. Additionally, the cavitation intensity declined with increasing percentages of organic liquids, as organic liquids reportedly generate lower collapse temperatures and reduce production of OH• [22]. By using organic fractions as 2.5 – 10%, there was no impact on the per-pass removal. By increasing the organic fraction to 20, 50 and 80%, the per-pass removal decreased by 60, 80 and 92% (Fig. 4b) – the final sulphur concentrations were 1746, 2128 and 2237 ppm (for 20, 50 and 80% respectively). In addition to the per-pass sulphur removal, it is also important to consider the actual sulphur removal rate at these conditions. The Eq. (7) was used to quantify the sulphur removal rate and the values were plotted in Fig. 3b.

(7) $$\mathrm{Sulphur}\;removal\;rate\left(\frac{\mu g}{\mathrm{hr}}\right)=\left[Q\left(LPH\right)\times 10^{-3}\right]\times \emptyset \times w_{s0}\left(mg\right)\times 10^{-6}$$

Here w_s0_ was the weight of sulphur in the holding tank at start of the experiment. It was possible to interpret the per-pass removal with organic volume fraction as:

(8) $$Per\text{-}pass\;removal\;\emptyset \times 10^{3}=28.5\times e^{\left[-0.03\times \theta \right]}$$

For sulphur removal rate (SRR, $\frac{\mu g}{\mathrm{hr}}$), was possible to interpret it as the following with organic fraction:

(9a) $$\mathrm{SRR}=2.8,\;when\;10<organic\;fraction\;\left(\%\right)<80$$

(9b) $$\mathrm{SRR}=0.85,\;when\;organic\;fraction\;\left(\%\right)\;\;2.5\%$$

The per-pass removal decreased by nearly 90% on increasing the organic fraction from 10 to 80%. However, the net sulphur removal rate exhibited a nearly constant trend across this same studied organic fraction. Despite a decrease in cavitation intensity and the per-pass removal (upon increase in organic fraction), the sulphur removal rate remained unaffected (see Fig. 4b). Upon comparison with results using a 12 mm vortex diode [8], both the per pass Sulphur removal factor and SRR are lower than the results obtained within this work which may be due to the organic volume fraction or organic phase properties, (n-octane is selected from [8] for comparison, as both n-octane and n-dodecane are aliphatic hydrocarbons with similar structures) – refer Table 3.

**Table 3:** Calculations for Sulphur removal rate and per-pass sulphur removal (basis C_0_ = 2500-ppm) for device Dev 2 at ΔP = 200-kPa, isothermal operation, *data compared with [8].

v_org_ (%)	v_organic_ (L)	Per-pass sulphur removal factor (ɸ x 10^3^)	w_S_ (ppm)	Sulphur removal rate × 10^−6^ (μg/hr)
2.5	0.14	32.1	130.6	1.12
10	0.55	32.1	522.5	4.46
20	1.10	13.1	1045.0	3.65
50	2.75	6.2	2612.5	4.34
80	4.40	2.6	4180.0	2.87
2.5*	0.3	10.50	90	0.61
10*	1.2	1.03	348	0.23

As discussed in our previous study, oxidation of dual ring thiophenes could not be realised in water-fuel systems and this trend continued for similar experiments with DBT at higher organic volume fractions (for water-fuel system) [16]. A plausible cause for this is that the diffusion timespan of hydroxyl radicals OH• is longer than their lifespan (10^−6^ s [26]). This means alternative sources of oxidising radicals are required. In the water-based study (without use of additives), water is assumed to play the role as dual oxidant/extractant, with production of OH• forming due to dissociation of entrapped water vapour at cavity collapse and subsequent extraction of the oxidised Sulphur species into the aqueous phase due to their increased polarity. However, oxidation of DBT was not possible with only water as an oxidant.

### 3.2 Removal of DBT

The optimised HC parameters identified in the thiophene study were applied for treatment of DBT from 2.5 to 80 v/v %. Similarly, to our previous work, no significant DBT removal occurred at any conditions. Therefore, additive based HC studies were required. Based on our previous work [16], 0.95 v/v % H_2_O_2_ oxidant and 6.25 v/v % HCOOH acid catalyst were used in this work. Additionally, regeneration of common extractants was attempted by mixing the same addition ratios in the HC configurations.

#### 3.2.1 With dosing of additives and catalyst

Studies for DBT removal with additives were attempted on three different experimental configurations (1, 2a, 2b and 3) at 4 different capacities with devices Dev 1, Dev 2 and Dev 3. An additional set of experiments was carried out with a smaller (1 LPM, throat diameter= 3 mm) vortex diode. After establishing a set-up that could operate without leaching, n-hexane was used as a medium in configuration 2a. Evaluation of dosing of additives has been reported to enhance Sulphur removal [27]. Two conditions of additive loading were compared for the open tank set-up, firstly with the total volume of additives added at t = 0 min (initial loading describing as Dosing 1) and spreading this volume with equal amounts being added at regular intervals (stepwise loading described as Dosing 2). The timing of intervals for addition of additives in the Dosing 2 technique were examined and described as Dosing 2a and 2b. A third technique of additive loading was utilised in the dodecane study below, essentially doubling the volume of additives by combining dosing 1 and 2 by utilising initial and stepwise addition (Dosing 3). Table 4 summarises the various loading techniques.

**Table 4:** Description of techniques of additive loading.

Description	Total additive loading	Additive Loading
Dosing 1	0.95 v/v % H_2_O_2_ + 6.25 v/v % HCOOH	100% addition @ t = 0 mins
Dosing 2a	0.95 v/v % H_2_O_2_ + 6.25 v/v % HCOOH	25 % addition @ t = 0, 10, 20, 30 mins
Dosing 2b	0.95 v/v % H_2_O_2_ + 6.25 v/v % HCOOH	25 % addition @ t = 0, 5, 10, 15 mins
Dosing 3	1.90 v/v % H_2_O_2_ + 12.50 v/v % HCOOH	50 % loading @ t = 0 mins, 12.5 % addition @ t = 5, 10, 15, 20 mins

In the hexane system, Dosing 1 saw an increase in DBT concentration, possibly due to evaporation of hexane increasing the concentration of DBT in solution. Although temperature was maintained at T = 20 ± 2 °C in the tank, hexane has a boiling point = 69 °C and some may have evaporated due to heat generated from the pump, heat of reaction or from the effects of cavitation. To assess whether the addition of large volumes of additives contributed to fuel stripping and whether possible rapid consumption of H_2_O_2_ was limiting DBT oxidation, stepwise addition was tested. The stepwise addition showed no significant change in the DBT concentration profiles suggesting there was evidence of a reaction occurring simultaneously with fuel stripping. To minimise the possibility of evaporation and ensure better mixing throughout the holding tank, the holding tank was altered from the open stainless-steel tank to a baffled beaker with cooling coil surrounding the pipe inlet to improve contact between the coolant and the reaction media (configuration 2b).

**Fig. 5:**
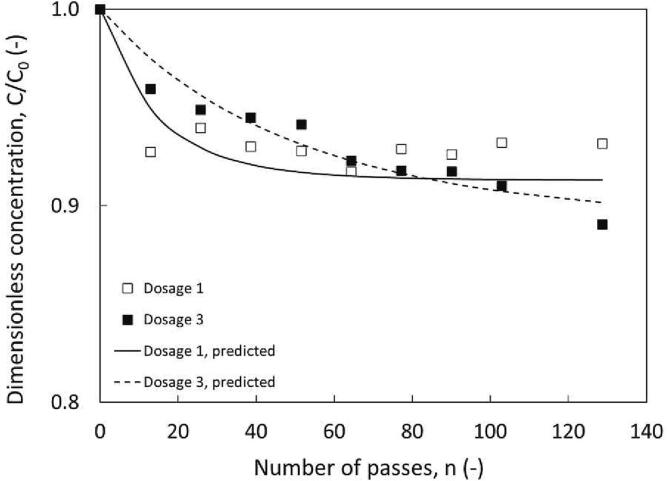
Influence of dosing on removal of DBT from dodecane, using configuration 2. ΔP = 200 kPa, Dev 3, (12 mm vortex diode), C_0, DBT_ = 300 ppm, v_total_ = 3.5 L, 0.95 v/v % H_2_O_2_, 6.25 v/v % HCOOH, ∅_0_ × 10^3^ and y (6.14 and 12 respectively) [dosage 1] and (2.25 and 9.6 respectively) [dosage 3].

Even under these experimental conditions with the improved cooling set-up, no significant change in DBT concentration was observed. The dosage interval was then reduced in Dosing 2b and performance was found to improve marginally. These results for hexane are included in the Supplementary Information (SI Fig. S1). To reduce the possibility of evaporation, n-hexane was replaced with n-dodecane, which did not appear to evaporate in the thiophene study. Dodecane has a higher boiling point, (216.2 °C) and the same set of experiments with was repeated with configuration 2b (Fig. 5). It is unlikely that dodecane will evaporate but a slight increase in concentration occurred after 30 min of circulation. This may be due to stripping of fuel entrapped within the aqueous droplets (i.e., oil in water in oil) to which this type of emulsion formation was discussed by Wu et al. 2021 for acoustic emulsification set-ups for bubble collapse at the liquid–liquid interface [28]. Experiments were attempted with Dev 2 but no degradation was achieved with visible phase separation occurring rapidly. To prevent phase separation, the larger 12 mm vortex diode was installed (Dev 3). As the larger scale of device demands higher flow rates, (i.e., Dev 1 = 4.5 LPM vs. Dev 3 = 18 LPM), to achieve the desired pressure drop, it was necessary to increase the set-up volume to 3.5 L. With these modifications oxidation was achieved with similar profiles obtained for initial and stepwise loading. To clarify whether the stepwise loading system was not being restricted by rapid consumption of the smaller quantities of H_2_O_2_ another dosing technique was established. This technique combined the initial loading and stepwise addition techniques, effectively doubling the volume of additives but only improving DBT oxidation from 6.8 − 11.0% (Fig. 5).

All experiments were completed at isothermal conditions (T = 20 ± 2 °C). Baradaran and Sadeghi reported 95.7% desulphurization (after extraction) for diesel and 95.8% removal for kerosene operating at 50 °C [14], [15]. To ensure striping is not occurring, preliminary experiments should be completed for each set-up. Our study so far has extended on HC-ODS literature for water-based systems and validated the design of a HC system with additives. Extension of the system to diesel allowed for a full comparison with HC publications with additive based systems [14], [15] and between model and actual fuels. These are discussed in the following.

#### 3.2.2 Influence of fuel type on removal of DBT

**Fig. 6:**
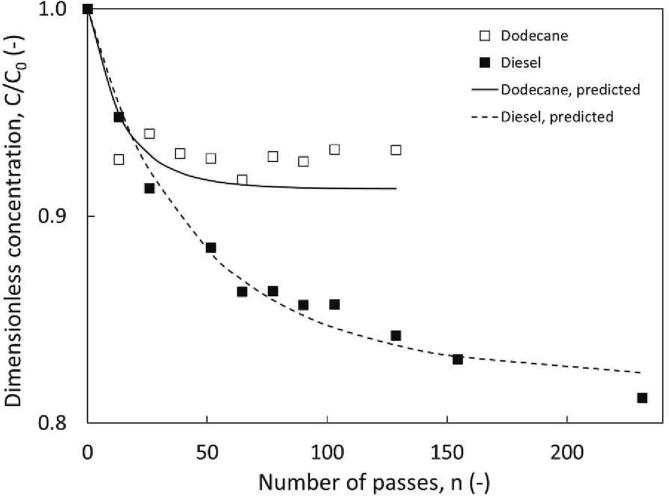
Influence of fuel type on DBT removal, using configuration 2. ΔP = 200 kPa, Dev 3, (12 mm vortex diode), C_0, DBT_ = 300 ppm, v_total_ = 3.5 L, 0.95 v/v % H_2_O_2_, 6.25 v/v % HCOOH, φ_0_ × 10^3^ and y = (6.14 and 12 respectively) [dodecane] and (4.25 and 6 respectively) [diesel].

Dodecane was used in this study to mimic diesel as the carbon number (C_12_) was within the range of diesel and thus diesel was selected for further experiments. Diesel was treated in Configuration 2b with device Dev 3_,_ and the same volumetric ratios of additives as stated previously. A comparison of dodecane and diesel is displayed in Fig. 6 with oxidation of DBT improving to 18.8% in the set-up circulating actual diesel. The composition of diesel was made up of various aliphatic and aromatic hydrocarbons, typically exhibiting a higher vapour pressure than n-dodecane, making diesel more susceptible to cavitation in a HC set-up.

Suryawanshi et al. commented that the polarity of the fuel may play a role determining the extent of interactions between the fuel and the aqueous phase [8]. This can be described by the dielectric constant ε which is approximately 2.2 for diesel and 2.01 for dodecane. They continued to emphasise, however, that the varying aliphatic and aromatic content of diesel made oxidation chemistry complex and difficult to predict a reaction mechanism [8]. The composition and polarity of diesel, coupled with a potentially higher number of cavitational events may contribute to the improved formation and stability of the fuel-water emulsion [23], [24]. Processing of real fuels may also benefit from an upgrading effect reducing their viscosity when exposed to cavitating conditions [29], [30] allowing for easier processing and mixing [31]. The viscosity of diesel is typically 1.9 to 3.7 mPa-s at 40 °C, and dodecane (used to simulate diesel) is 0.8498 mPa-s at 25 °C [32]. This upgrading effect may not be as pronounced in the dodecane set-up as significant degradation or conversion of the C_12_ carbon chain [33].

**Fig. 7:**
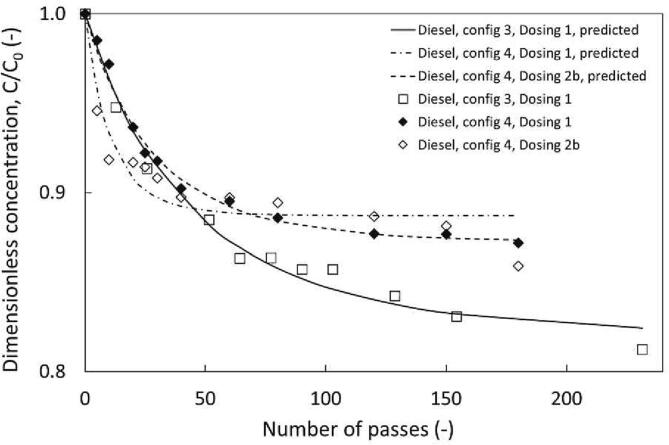
Influence of configuration on DBT removal, using configuration 2b and 3. ΔP = 200 kPa, Dev 3*,* configuration 2 = 3.5 L, v, configuration 3 = 9 L, (12 mm vortex diode), C_0, DBT_ = 300 ppm, 0.95 v/v H_2_O_2_, 6.25 v/v % HCOOH*,*∅ × 10^3^ and y = (4.25 and 6 respectively) [config 2, Dosing 1], (10.81 and 9.33 respectively) [config 3, Dosing 1], (4.62 and 8.3 respectively) [config 3, Dosing 2b].

Development of the HC configuration has shown increasing the degree of physical mixing can encourage emulsification ensuring circulation of both phases through the cavitation device. By increasing the device scale and corresponding required flow rate the extent of oxidation was enhanced. To achieve further physical enhancement, Dev 3 was installed on configuration 3 with a capacity of 9 L, with bottom suction pump inlet to prevent stagnation of the denser aqueous phase in the holding tank. Degradation profiles of DBT in Configurations 2 and 3 are compared in Fig. 7 which shows that despite the much larger capacity of Configuration 3, there was little deviation in the observed oxidation between set-ups. Generally, in the treatment of organic pollutants in water-based systems, increasing the scale of cavitation device portrays a diminishing degradation trend [10].

For liquid–liquid systems, the formation of a fine emulsion was essential to overcome mass transfer limitations. As the results of this study suggests the role of cavitation was primarily physical [18], [34]. The impact of up-scaling the process capacity and scale of cavitation device may not diminish degradation in the same manner as single-phase processes which depend on physiochemical enhancement by cavitation (assuming the intensity of mixing is maintained). In the HC-ODS process with additives, the contribution of chemical enhancement is unclear, but significant physical enhancement is essential for overcoming the mass transfer limitations in ODS. The comparison of devices in the water-fuel study for Thiophene treatment show that Dev 1 performed the best of the devices tested but the larger vortex diode, Dev 3 achieved the best results in the additive based study. This may be explained by the expected degradation/oxidation mechanisms, as degradation in the water-based system is assumed to be a result of physiochemical transformations generated by cavitation.

When comparing device performance in the additive based study, installation of Dev 3 was required to ensure adequate physical transformations were produced in the medium. As chemical transformations are induced by oxidising species produced from the reaction of H_2_O_2_ and HCOOH [16]. The required chemical contribution from cavitation may be reduced providing a possible explanation to why physical enhancement was dominant in this particular set-up. Importantly, the comparison of device type showed that the thiophene degradation trend for Dev 3 was almost the same as the linear orifice Dev 2, despite operating at higher flow rates and capacities. This suggested the suitability of vortex-based devices for additive based HC-ODS. The influence of vortex based devices in liquid–liquid systems has been discussed in recent publications by Thaker and Ranade, showing that vortex-based cavitation devices have been effective for reducing the droplet diameter in a single pass through the device [35], [36], [37]. Applying this in ODS, vortex-based devices may effectively improve the fineness of the fuel emulsion increasing the interfacial area and improving oxidation. This effect is reflected in Fig. 7 which shows a similar extent of DBT oxidation after 180 passes for the 3.5-L and 9-L capacity set-ups with 16.1% and 14.8% DBT oxidation respectively, suggesting that the physical transformations induced by vortex based cavitation may be upheld at increased set-up volumes.

### 3.3 Comparison with other additives, fuels, and techniques

**Fig. 8:**
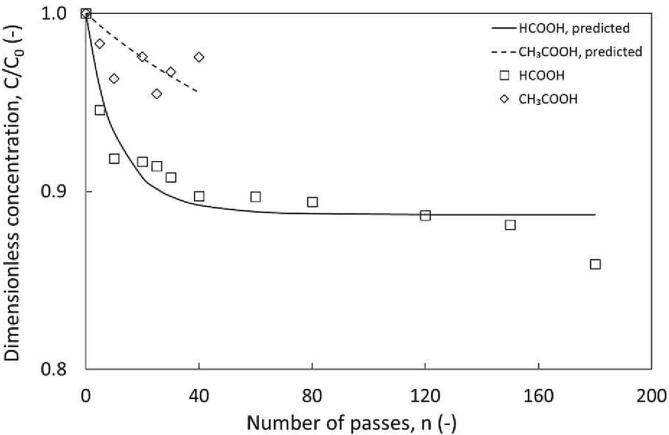
Influence of acid catalyst on DBT removal, using configuration 3. ΔP = 200 kPa, Dev 3 (12 mm vortex diode), v_total_ = 9 L, C_0, DBT_ = 300 ppm, 0.95 v/v H_2_O_2_, 6.25 v/v % HCOOH or CH_3_COOH, ∅ × 10^3^ and y = (10.81 and 9.33 respectively) [HCOOH] and (1.42 and 8.3 respectively) [CH_3_COOH].

Our previous study focused on identifying the optimum ratios for H_2_O_2_ beaker scale ODS with model fuels. In this study, we have extended this to HC. Our previous work compared the type of acid catalyst, comparing HCOOH and CH_3_COOH in an AC set-up with n-dodecane as the medium. HCOOH achieved 68.5% DBT oxidation compared to 11.05% with CH_3_COOH under the same conditions. A similar result was obtained in the HC study on Configuration 3, (Fig. 8) with a slight decrease in DBT concentration with CH_3_COOH which quickly stabilised with no further oxidation achieved beyond this point. This may occur because peroxyformic acid portrays a higher reactivity towards the organic acid interface [38]. This allowed us to confirm that the trends are somewhat transferrable from AC to HC set-ups and that a reduction of the extent of oxidation may be limited by the system volume or properties of the fuel.

**Fig. 9:**
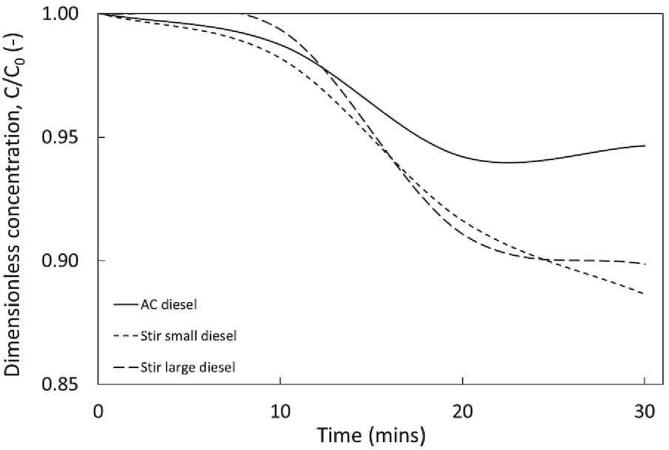
Influence of smaller scale testing on diesel and C_0_, _DBT_ = 300 ppm, v_stir,small_ = 40 mL, v_stir, large_ = 1 L, v_AC_ = 40 mL. Stirring rate = 350 RPM (small) and 1000 RPM (large). Acoustic cavitation parameters amplitude = 80%, 50 % Duty cycle, frequency = 20 kHz.

It will be useful to compare AC conditions identified in our previous study for actual fuels. Results show there was no impact on DBT oxidation for two scales of stirred systems (v_, small_ = 40 mL and v_, large_ = 1 L) whereas AC (v_,AC_ = 40 mL) performed the worst of all the processes implemented within this study, (Fig. 9). A plausible explanation for this may be due to the evaporation of lighter compounds in diesel when subjected to ultrasonication, increasing the concentration of the more viscous compounds in the fuel, and restricting mass transfer [29], [39].

Similar experiments were conducted for kerosene and petrol. Kerosene showed no significant removal but interpretation of the results was extremely complex due to overlapping signals on the HPLC-UV-FLD. HC treatment of both fuels was also attempted but the analytical complexities increased, and the results could not be interpreted. The more volatile petrol saw dramatically increasing DBT concentrations, showing similar behaviour to n-hexane in the HC set-up (as somewhat expected as n-hexane carbon number mimics that of petrol). When comparing these values to HC the benefits of easier scalability and reduced energy consumption are apparent. Sulphur removal per energy expense (mg/J) was calculated using Equation (10) as:

(10) $$Y\left(\frac{\mathrm{mg}}{J}\right)=\frac{w_{\mathrm{DBT},rem}}{E}$$

Where, Y is the mass of DBT oxidised per unit energy expenditure (mg/J), w_,DBT,rem_ is the mass of DBT removed (mg) and E is the energy expenditure. Energy consumption for AC was read from the ultrasonic generator. For HC, energy consumption was calculated by the following equation:

(11) E=ΔPQt

AC for diesel was the most energy intensive, recorded at 1.7 × 10^−5^ mg/J compared to stirring, which performance improved with scale from 4.2 × 10^−5^ mg/J (small) to 1.1 × 10^−3^ mg/J (large). HC on Configuration 4, performed the best in the diesel study, achieving removal of 1.2 × 10^−3^ mg/J.

It is important to note, that oxidation achieved for n-dodecane in an AC set-up achieved 91% DBT oxidation [16] whereas an upper limit of 20% oxidation could be achieved in the HC set-ups. The extent of oxidation for diesel, however, improved in HC set-ups compared to AC and stirring. This suggests that HC may be more suitable for treatment of actual fuels. Droplet sizes were not measured for the liquid–liquid systems considered in this work. However, these measurements have been carried out for oil in water emulsions by Thaker and Ranade [37]. This study clearly demonstrates that hydrodynamic cavitation generates droplets smaller than 10 µm and therefore greatly enhances liquid–liquid interfacial area.

## 4 Conclusions

This study was separated into two parts focusing on treatment of fuels with HC in water and additive systems respectively. The first part examined Thiophene degradation, identifying optimum process parameters, influence of scale of device and device type. Aeration was utilised as an intensification strategy for Thiophene removal. Building on this, attempts were to oxidise DBT without any external catalyst and oxidant and this was not successful. Therefore, external oxidant (H_2_O_2_) and catalyst (HCOOH) were used in the second part. The concentrations of H_2_O_2_ and HCOOH were set to 0.95 v/v % and 6.25 v/v% respectively based on our previous work. Regeneration of extractants as well as treatment of model and actual fuels in HC systems were investigated with these additives. The key conclusions from the study are as follows:

• Initial Thiophene concentration did not significantly influence the extent of degradation
• Increasing the organic volume from 2.5 to 80 v/v % reduced the per pass removal from 32.1 to 13.1. However, overall rate of removal remained almost the same over the whole range of organic volume fraction
• Aeration at 1.9 vvm enhanced Thiophene removal by 3.6 times for the linear flow cavitation device
• Dev 1 (6-mm vortex diode), smallest scale of operation performed best, as observed with the per-pass removal factor of the three devices for Thiophene removal
• Stripping of fuels (n-hexane and petrol) was significant and proper temperature control and cooling was essential to quantify desulphurization
• Dosing strategy of additives showed little impact on extent of DBT oxidation
• The results indicate that HC’s contribution in enhancing ODS performance is primarily physical (by enhancing mass transfer via reducing droplet size of emulsions)
• HC was found to remove 20% DBT from diesel that was more than that observed with AC and stirred set-up

These results and the results presented here show the potential of using HC for intensifying ODS with or without external catalyst and oxidants.

## CRediT authorship contribution statement

**Peter Delaney:** Investigation, Data curation, Validation. **Varaha P. Sarvothaman:** Investigation, Methodology, Data curation, Validation. **Sanjay Nagarajan:** Validation. **David Rooney:** Supervision. **Peter K.J. Robertson:** Funding acquisition, Supervision. **Vivek V. Ranade:** Conceptualization, Funding acquisition, Supervision, Writing – review & editing.

## Declaration of Competing Interest

The authors declare that they have no known competing financial interests or personal relationships that could have appeared to influence the work reported in this paper.

## Data Availability

Data will be made available on request.
